# Association between the metabolic score for insulin resistance and osteoarthritis prevalence: A cross-sectional population-based study

**DOI:** 10.1097/MD.0000000000044850

**Published:** 2025-10-31

**Authors:** Yanghua Weng, Hongdong Yang, Langming Li, Shuchi Lv, Guibin Chen

**Affiliations:** a Department of Orthopedics, Dongguan Humen Hospital, Dongguan, China.

**Keywords:** cross-sectional study, insulin resistance, metabolic score for insulin resistance, NHANES, osteoarthritis

## Abstract

Insulin resistance (IR) is associated with osteoarthritis (OA). The metabolic score for insulin resistance (METS-IR) is a tool for evaluating IR without direct insulin measurement, demonstrating potential across many metabolic disorders. Nonetheless, its association with OA remains ambiguous. The National Health and Nutrition Examination Survey 2011–2018 data set was examined in this cross-sectional analysis. METS-IR was computed using widespread metabolic markers, and OA was determined by self-reported physician diagnosis. The association between METS-IR and OA was assessed using logistic regression models that controlled for comorbidities, lifestyle, and demographics. METS-IR was assessed both continuously and by quartiles. Restricted cubic splines were used to examine dose–response relationships. To verify robustness, sensitivity and subgroup analyses were conducted. OA was diagnosed in 1085 of the 8799 individuals who were surveyed. A higher METS-IR was strongly correlated with elevated chances of OA (OR = 1.02; 95% CI: 1.01–1.03). The prevalence of OA was significantly higher among individuals in the highest METS-IR quartile than in the lowest quartile (OR = 1.73; 95% CI: 1.21–2.45). Restricted cubic spline analysis indicated a linear correlation. Subgroup and sensitivity studies validated the robustness and generalizability of the results. In this cross-sectional study of a US population, higher METS-IR levels were linearly and positively associated with OA prevalence. As a surrogate indicator of IR derived from routine metabolic parameters, METS-IR may serve as a valuable epidemiological tool for identifying individuals at increased risk of OA.

## 1. Introduction

Osteoarthritis (OA) is a persistent degenerative joint disorder marked by ongoing synovial inflammation, subchondral bone remodeling, and deterioration of articular cartilage.^[1]^ Clinically, it manifests as joint pain, stiffness, limited mobility, and functional impairment.^[2,3]^ OA is the most common arthropathy in middle aged and older persons, with prevalence markedly rising with age and linked to several medical conditions.^[4,5]^ Research on the global burden of disease reveals that the incidence of OA has steadily risen in recent decades, emerging as a principal cause of disability globally.^[6–8]^ This condition severely compromises the quality of life in the elderly population and imposes a substantial burden on healthcare systems and society.^[9,10]^ Although OA was traditionally viewed as a degenerative condition primarily driven by mechanical stress, recent studies have highlighted its etiological heterogeneity, with metabolic dysregulation playing a crucial role in its pathogenesis.^[11,12]^ Given the high prevalence and irreversible progression of OA, identifying metabolic indicators closely associated with the presence of OA provides valuable epidemiological insights for early screening and precision prevention in populations with a high likelihood of disease.

Insulin resistance (IR), a fundamental characteristic of metabolic syndrome, is widely acknowledged as a crucial pathogenic connection among obesity, systemic inflammation, and joint deterioration.^[13]^ Through a range of metabolic and inflammatory alterations, IR may contribute to the development and progression of OA, thereby promoting the emergence of a metabolically driven OA phenotype.^[14,15]^ The metabolic score for insulin resistance (METS-IR), calculated as (Ln[2 × FPG + TG] × BMI)/Ln (HDL-C) where FPG denotes fasting plasma glucose, TG triglycerides, BMI body mass index, and HDL-C high-density lipoprotein cholesterol, has recently been identified as a surrogate marker for IR that does not require direct insulin measurements.^[16]^ As a composite index integrating glucose and lipid metabolism with adiposity, METS-IR may better capture the systemic metabolic dysfunction underlying low-grade inflammation, lipid accumulation, and chondrocyte metabolic dysregulation – key processes implicated in OA. It has shown strong validity and stability in several studies.^[17–19]^ METS-IR is facile to calculate, economical, and suitable for general health assessments. It has been demonstrated to be significantly associated with several metabolism-related disorders, including chronic kidney disease, type 2 diabetes, and negative cardiovascular outcomes.^[20–23]^ Moreover, prior studies have demonstrated a significant association between heightened METS-IR levels and diminished bone mineral density, elevated FRAX scores, and a higher prevalence of fractures among nondiabetic persons in the United States, indicating its possible involvement in bone metabolism problems.^[24]^ However, whether a relationship exists between METS-IR and OA remains unclear, as systematic cross-sectional evidence is still lacking. Against this background, the current study employed nationally representative cross-sectional data to examine the association between METS-IR levels and the prevalence of OA. The aim was to assess the potential of METS-IR in identifying individuals with OA in the general population, and to provide foundational data and theoretical support for future research on risk stratification, pathophysiological mechanisms, and targeted prevention of metabolically driven OA.

### 1.1. Survey description and study population

This study used data from the 2011–2018 National Health and Nutrition Examination Survey (NHANES). The CDC’s National Center for Health Statistics sponsors NHANES, a cross-sectional survey. Stratified, multistage probability sampling yields nationally representative estimates for noninstitutionalized civilian US population. NCHS Research Ethics Review Board authorized the study method, and all subjects gave written informed permission.

Participants were eligible for inclusion if they: were aged 20 years or older; completed the arthritis-related questionnaire; and had available laboratory and physical examination data required to calculate the METS-IR score. Individuals with missing values in any of these key variables were excluded. After applying these criteria, a total of eligible adult participants were included in the final analysis.

### 1.2. Calculation of METS-IR

The METS-IR is a surrogate index derived from routine metabolic parameters to assess an individual’s level of IR. As reported in previous studies,^[25–27]^ the calculation formula is as follows:


$$\begin{array}{l} \begin{array}{lll} METS\text{-}IR= & ln\left[2\times FPG\;\left(mg/dL\right)+TG\left(mg/dL\right)\right]\; & \times BMI/ln\left[HDL\text{-}C\left(mg/dL\right)\right]\; \end{array} \end{array}$$

where FPG denotes fasting plasma glucose, TG is triglycerides, BMI refers to body mass index, and HDL-C represents high-density lipoprotein cholesterol. All biochemical parameters were measured from venous blood samples collected after at least 8.5 hours of fasting. Measurements were performed using an enzymatic colorimetric method with an automated clinical chemistry analyzer.

### 1.3. Definition of osteoarthritis

OA was diagnosed based on participants’ self-reported data during the questionnaire interview. Respondents were first inquired if they had ever received a diagnosis of arthritis from a healthcare provider. Upon receiving a “yes” response, participants were subsequently prompted to identify the specific type of arthritis. Individuals who indicated “osteoarthritis” were categorized as having OA. This self-reported method has been widely adopted in NHANES-related studies, and previous research has demonstrated its acceptable reliability and validity, supporting its consistency and feasibility in large-scale epidemiological investigations.^[28–30]^

### 1.4. Covariates

To comprehensively address potential confounding variables in the association between METS-IR and OA, multiple covariates, encompassing demographic characteristics, lifestyle habits, and chronic health conditions, were integrated into the multivariable logistic regression models to enhance the clarity and scientific rigor of the findings. Demographic characteristics were age, sex, race, educational level, and poverty income ratio. According to established norms, the poverty income ratio classed households as low income (<1), middle income (1–3), or high income (≥3). Lifestyle habits including smoking status, drinking, and physical activity levels. Smoking is characterized as having consumed over 100 cigarettes in one’s lifetime. Drinking was characterized as any usage of alcohol within the preceding 12 months. Insufficient physical activity is defined as total weekly metabolic equivalents of fewer than 600 minutes, in accordance with World Health Organization standards. Chronic disease-related covariates included self-reported physician diagnoses of diabetes, hypertension, coronary artery disease, and chronic kidney disease. These covariates were selected as potential shared risk factors for both OA and metabolic disorders. Their inclusion aimed to minimize residual confounding, enhance model robustness, and ensure a more reliable and valid estimation of the independent association between METS-IR and OA.

### 1.5. Statistical analysis

This research employed continuous cross-sectional data from the NHANES 2011–2018 cycles to perform statistical analysis on people who satisfied the inclusion criteria. Descriptive statistics were initially employed to assess demographic and clinical features between people with and without OA. Multivariable logistic regression models with 3 levels of adjustment were created in order to examine the independent association between METS-IR and the prevalence of OA: model 1 was left unadjusted, model 2 obtained demographic factors into consideration, and model 3 added adjustments for lifestyle and health-related covariates. METS-IR was evaluated as both a continuous and categorical variable, utilizing quartiles to examine possible dose–response associations across varying exposure levels. A restricted cubic spline (RCS) regression model was utilized to investigate possible nonlinear dose–response associations between METS-IR and the prevalence of OA. Subgroup analyses were performed stratified by demographic factors, with interaction terms tested to evaluate heterogeneity across subgroups. To ensure robustness, sensitivity analyses were performed by eliminating individuals having METS-IR values over ±3 standard deviation from the mean and reestimating regression models. All analyses were done with R software (version 4.2.3), and *P*-values < .05 were thought to be significant.

## 2. Results

### 2.1. Baseline characteristics

**Figure 1.**
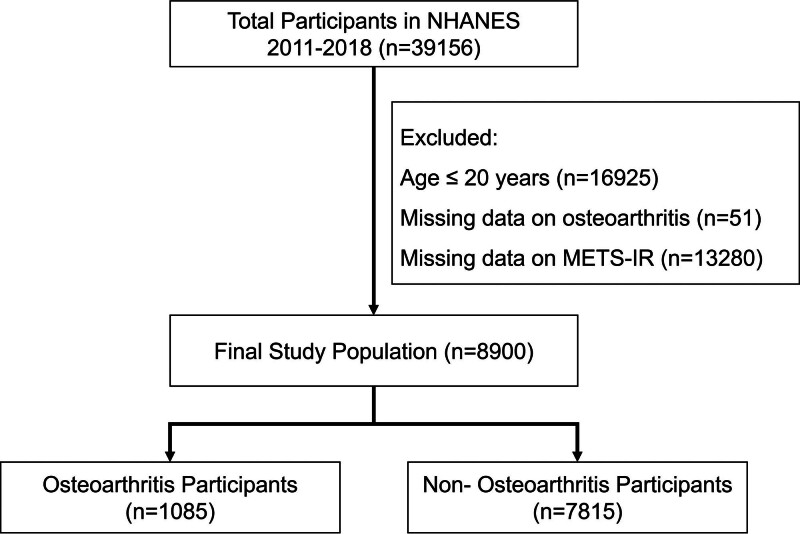
Flowchart of study population selection. METS-IR = metabolic score for insulin resistance, NHANES = National Health and Nutrition Examination Survey.

According to data from the NHANES 2011–2018 cycles and the established inclusion and exclusion criteria, a total of 8799 persons aged 20 years and older were incorporated into the final analysis. Of the participants, 1085 self-reported a physician-diagnosed OA, while the rest 7814 were categorized as the non-OA group (Fig. 1). Table 1 delineates the comparison of baseline characteristics between the 2 cohorts. Marked disparities were noted in many demographic and clinical factors. In comparison to the non-OA group, those with OA were more likely to be non-Hispanic White, older, and female on average. The average METS-IR score was markedly elevated in the OA group compared to the non-OA group, indicating that IR may contribute to the prevalence of OA and offering initial evidence for further association research.

**Table 1:** Descriptive characteristics of the analytical sample.

Characteristic	Group	Overall	Non-osteoarthritis	Osteoarthritis	*P*-value
n		8900	7815	1085	
Age (%)	<50	4479 (50.3)	4273 (54.7)	206 (19.0)	<.001
	>50	4421 (49.7)	3542 (45.3)	879 (81.0)	
Sex (%)	Female	4595 (51.6)	3894 (49.8)	701 (64.6)	<.001
	Male	4305 (48.4)	3921 (50.2)	384 (35.4)	
Race (%)	Mexican American	1231 (13.8)	1136 (14.5)	95 (8.8)	<.001
	Non-Hispanic Black	1845 (20.7)	1673 (21.4)	172 (15.9)	
	Non-Hispanic White	3374 (37.9)	2750 (35.2)	624 (57.5)	
	Others	2450 (27.5)	2256 (28.9)	194 (17.9)	
Education level (%)	Under high school	1981 (22.3)	1768 (22.6)	213 (19.6)	.125
	Above high school	4969 (55.8)	4335 (55.5)	634 (58.4)	
	High school or equivalent	1947 (21.9)	1709 (21.9)	238 (21.9)	
	No record	3 (0.0)	3 (0.0)	0 (0.0)	
PIR (%)	<1	1709 (21.3)	1542 (21.9)	167 (17.0)	.002
	1–3	3366 (42.0)	2929 (41.6)	437 (44.4)	
	>3	2943 (36.7)	2563 (36.4)	380 (38.6)	
Smoke (%)	No	5055 (56.8)	4536 (58.0)	519 (47.8)	<.001
	Yes	3837 (43.1)	3271 (41.9)	566 (52.2)	
	No record	8 (0.1)	8 (0.1)	0 (0.0)	
Drink (%)	No	1936 (23.5)	1688 (23.5)	248 (23.9)	.771
	Yes	6293 (76.4)	5503 (76.5)	790 (76.1)	
	No record	3 (0.0)	3 (0.0)	0 (0.0)	
Diabetes (%)	No	7378 (82.9)	6589 (84.3)	789 (72.7)	<.001
	Yes	1272 (14.3)	1022 (13.1)	250 (23.0)	
	No record	250 (2.8)	204 (2.6)	46 (4.2)	
Activity status (%)	Active	4701 (52.8)	4249 (54.4)	452 (41.7)	<.001
	Inactive	4199 (47.2)	3566 (45.6)	633 (58.3)	
CAD (%)	No	8574 (96.3)	7566 (96.8)	1008 (92.9)	<.001
	Yes	314 (3.5)	239 (3.1)	75 (6.9)	
	No record	12 (0.1)	10 (0.1)	2 (0.2)	
CKD (%)	No	8554 (96.1)	7553 (96.6)	1001 (92.3)	<.001
	Yes	335 (3.8)	252 (3.2)	83 (7.6)	
	No record	11 (0.1)	10 (0.1)	1 (0.1)	
Hypertension (%)	No	5525 (62.1)	5120 (65.5)	405 (37.3)	<.001
	Yes	3362 (37.8)	2685 (34.4)	677 (62.4)	
	No record	13 (0.1)	10 (0.1)	3 (0.3)	
TG (mean [SD], mg/dL)		119.51 (106.78)	118.28 (108.66)	128.33 (91.74)	.004
HDL (mean [SD], mg/dL)		53.97 (16.06)	53.67 (15.74)	56.13 (18.09)	<.001
BMI (mean [SD], kg/m^2^)		29.37 (7.11)	29.07 (6.93)	31.54 (7.97)	<.001
FPG (mean [SD], mg/dL)		110.79 (36.47)	110.06 (36.05)	116.02 (38.98)	<.001
METS-IR (mean [SD])		43.66 (12.89)	43.21 (12.62)	46.98 (14.24)	<.001
METS-IR (%)	Q1) < 34,42)	2225 (25.0)	2022 (25.9)	203 (18.7)	<.001
	Q2 (34.42–41.64)	2225 (25.0)	1981 (25.3)	244 (22.5)	
	Q3 (41.64–50.44)	2225 (25.0)	1957 (25.0)	268 (24.7)	
	Q4 (>50.44)	2225 (25.0)	1855 (23.7)	370 (34.1)	

Mean (SD) for continuous variables, % for categorical variables.

BMI = body mass index, CAD = coronary artery disease, CKD = chronic kidney disease, FPG = fasting plasma glucose, HDL = high-density lipoprotein, METS-IR = metabolic score for insulin resistance, PIR = poverty index ratio, SD = standard deviation, TG = triglyceride.

### 2.2. Association between METS-IR and osteoarthritis prevalence

**Figure 2.**
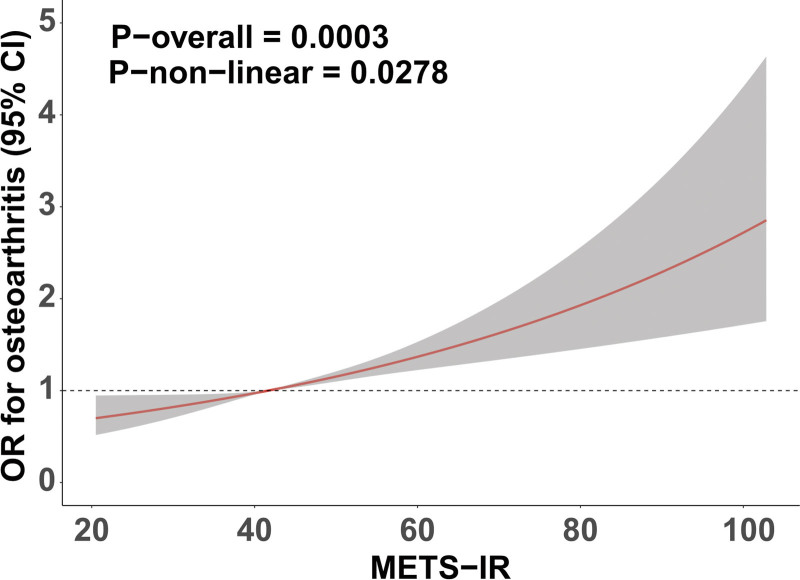
RCS model illustrating the association between the METS-IR and OA prevalence. Adjusted for age, sex, race, educational level, PIR, smoke, drink, activity status, diabetes, CKD, and hypercholesterolemia. CI = confidence interval, CKD = chronic kidney disease, METS-IR = metabolic score for insulin resistance, OA = osteoarthritis, OR = odds ratio, PIR = poverty index ratio, RCS = restricted cubic spline.

The outcomes of the multivariable logistic regression analysis are displayed in Table 2. Upon including METS-IR into the model as a continuous variable, a positive correlation with OA prevalence was identified. In the unadjusted model, a 1-unit increase in METS-IR associated with a 2% heightened likelihood of OA (OR = 1.02, 95% CI: 1.01–1.02). The association maintained statistical significance after controlling for demographic characteristics in model 2 and when adjusted for lifestyle and chronic disease variables in model 3 (OR = 1.02, 95% CI: 1.01–1.03), illustrating the independent and resilient nature of the association between METS-IR and OA. When METS-IR was assessed categorically, compared with the lowest quartile (Q1), the odds ratios for the second (Q2: OR = 1.32, 95% CI: 0.93–1.87) and third quartiles (Q3: OR = 1.27, 95% CI: 0.92–1.75) indicated nonsignificant positive trends. Although not statistically significant, these findings suggest a potential dose–response pattern, which became significant in the highest quartile (Q4: OR = 1.72, 95% CI: 1.21–2.45). Furthermore, RCS analysis revealed a linear association between METS-IR and the prevalence of OA (Fig. 2). This indicates a consistently rising linear association between elevated METS-IR levels and the probability of developing OA.

**Table 2:** Association between METS-IR and osteoarthritis prevalence.

		Model 1 OR (95% CI) *P*-value	Model 2 OR (95% CI) *P*-value	Model 3 OR (95% CI) *P*-value
Osteoarthritis	METS-IR	1.02 (1.01, 1.02) <.001	1.02 (1.02, 1.03) <.001	1.02 (1.01, 1.03) <.001
	Q1	[Reference]	[Reference]	[Reference]
	Q2	1.27 (0.96, 1.69) .100	1.27 (0.94, 1.72) .120	1.32 (0.93, 1.87) .120
	Q3	1.22 (0.93, 1.58) .150	1.30 (0.96, 1.75) .087	1.27 (0.92, 1.75) .150
	Q4	1.79 (1.36, 2.35) <.001	2.00 (1.49, 2.67) <.001	1.72 (1.21, 2.45) .003
	*P* for trend	<.001	<.001	.002

Model 1: no covariates adjusted; model 2: adjusted for age, sex, and race; model 3: adjusted for age, sex, race, educational level, PIR, smoke, drink, activity status, diabetes, CKD, and hypercholesterolemia.

CI = confidence interval, CKD = chronic kidney disease, METS-IR = metabolic score for insulin resistance, OR = odds ratio, PIR = poverty index ratio, Q = quartiles.

### 2.3. Subgroup and sensitivity analyses

To assess the consistency and intensity of the relationship between METS-IR and OA, demographic subgroup analyses were conducted (Table 3). The results demonstrated a mostly advantageous association between METS-IR and OA prevalence across all categories, with no statistically significant interactions detected. These findings suggest that the observed association is stable and broadly applicable across different population strata. Furthermore, a sensitivity analysis was conducted by excluding participants whose METS-IR values were more than ±3 SD from the mean in order to assess the impact of extreme values. After exclusion, 1062 individuals with OA and 7739 without OA were included in the reanalysis (Table S1, Supplemental Digital Content, https://links.lww.com/MD/Q439). The results remained consistent with the primary analysis, with only minor variations in effect estimates, further confirming the robustness of the association and its resistance to outlier influence.

**Table 3:** Subgroup analysis between METS-IR and osteoarthritis.

Characteristic	Group	OR (95% CI) *P*-value	*P* for interaction
Age	<50	1.02 (1.01, 1.04) .001	.072
	>50	1.01 (1.01, 1.02) .002	
Sex	Male	1.02 (1.00, 1.03) .010	>.900
	Female	1.02 (1.01, 1.03) <.001	
Race	Mexican American	1.02 (1.00, 1.04) .055	.200
	Non-Hispanic Black	1.02 (1.00, 1.03) .021	
	Non-Hispanic White	1.01 (1.00, 1.02) .005	
	Others	1.04 (1.02, 1.06) <.001	
PIR	<1	1.02 (1.00, 1.03) .037	>.900
	1–3	1.02 (1.01, 1.03) .004	
	>3	1.02 (1.01, 1.03) .003	

CI = confidence interval, METS-IR = metabolic score for insulin resistance, OR = odds ratio, PIR = poverty index ratio.

## 3. Discussion

Using nationally representative data from the NHANES 2011–2018 cycles, this study found a significant positive association between the prevalence of OA and elevated METS-IR levels. This association remained robust after adjustment for multiple covariates, showed a dose-dependent pattern, and was consistently observed across various subgroups. These findings suggest that METS-IR, a composite index derived from routine metabolic parameters, may serve as a promising epidemiological marker for identifying individuals at higher risk of OA in the context of IR. Owing to its simplicity, cost-effectiveness, and ease of implementation, METS-IR holds potential clinical utility for population-level screening and individualized prevention strategies for OA.

With advancing understanding of OA pathogenesis, the traditional model of structurally driven degeneration centered on mechanical wear has gradually evolved into a multifactorial paradigm characterized by the interplay of mechanical loading, metabolic dysregulation, and inflammation.^[12,31,32]^ Within this framework, metabolic disturbances – particularly IR – have increasingly been recognized as key intrinsic drivers of OA progression.^[12,33,34]^ Previous studies have primarily focused on individual metabolic factors such as obesity, hyperglycemia, and dyslipidemia in relation to OA, whereas investigations into IR – the core mechanism of metabolic syndrome – remain limited.^[35–37]^ Some evidence suggests that IR may contribute to OA onset by inducing chronic inflammation and disrupting chondrocyte metabolic homeostasis.^[38,39]^ However, traditional indices of IR such as HOMA-IR require fasting insulin measurements, which restricts their use in large-scale population-based studies and may be influenced by laboratory variability.^[40,41]^ In contrast, METS-IR is derived from readily available clinical parameters and does not rely on insulin assays. Prior investigations using HOMA-IR have reported that higher levels of IR are associated with an increased prevalence of OA, supporting the role of impaired insulin signaling in joint degeneration.^[42,43]^ In the present study, METS-IR was significantly associated with OA prevalence as a continuous variable, and a dose–response trend was observed across quartiles, indicating that greater metabolic burden correlates with increased OA risk. The RCS analysis further revealed a linear association without an apparent threshold, suggesting a consistent and sustained risk gradient across the metabolic spectrum. Subgroup and sensitivity analyses confirmed the robustness and generalizability of this association across diverse populations, underscoring the potential utility of METS-IR in stratified risk management for OA. In conclusion, METS-IR, a comprehensive score based on routine metabolic parameters, demonstrated a stable and significant positive association with the OA prevalence in this study. These findings offer epidemiological data corroborating the association between metabolic state and joint health, indicating that METS-IR may function as a straightforward and accessible supplementary tool for the initial screening and early detection of persons at elevated risk for OA. More longitudinal investigations are necessary to clarify the temporal link and stratified risk value of METS-IR in the onset and advancement of OA.

From a mechanistic standpoint, the IR state reflected by the METS-IR index may facilitate the development and progress of OA through a network of interrelated biological processes. As a hallmark of metabolic dysfunction, IR is closely associated with systemic low-grade inflammation and disruption of local joint homeostasis, potentially leading to dysregulated synovial immune responses and accumulation of pro-inflammatory mediators, thereby accelerating cartilage damage.^[44–47]^ Concurrently, IR is often accompanied by disturbances in lipid metabolism, including dysregulated adipose tissue signaling and lipid accumulation, which may increase the sensitivity of joint cells to external stressors and impair stress response pathways, ultimately undermining the structural and functional integrity of joint tissues.^[34,48–50]^ In addition, phenotypic features commonly observed in IR – such as increased body weight and dyslipidemia – are linked to heightened oxidative stress and the accumulation of metabolic byproducts within joint tissues, reducing the regenerative capacity of cartilage and subchondral bone.^[38,51,52]^ Structurally, IR may also interfere with the remodeling balance of subchondral bone, disrupt the biomechanical coupling between cartilage and bone, and lead to abnormal stress transmission, thereby promoting degenerative changes in joint architecture.^[53–55]^ These complex and overlapping mechanisms collectively provide a plausible biological basis for the observed association between elevated METS-IR levels and increased OA risk. Although this study provides preliminary epidemiological evidence supporting this relationship, the underlying molecular pathways were not directly investigated and warrant further exploration through experimental models, prospective cohort studies, and tissue-level functional analyses to clarify causal relationships and identify key regulatory mechanisms.

This study offers several notable strengths. First, the data were derived from the NHANES, a nationally representative survey of the US population, ensuring strong external validity and rigorous quality control. Second, METS-IR is calculated using routine clinical measurements, making it readily applicable in both clinical settings and primary public health practice. Third, the study employed a comprehensive statistical framework, including multivariable logistic regression, quartile-based trend analysis, RCS modeling, and both subgroup and sensitivity analyses, thereby minimizing confounding bias and enhancing the robustness and interpretability of the findings. Nevertheless, it is imperative to recognize a number of restrictions. As a cross-sectional study, causal inference cannot be established, necessitating confirmation through prospective cohort studies. Additionally, OA diagnosis was based on self-reports, which, although commonly used in NHANES, may introduce misclassification or recall bias. The study also lacked information on the anatomical sites and severity of OA, limiting the ability to analyze distinct OA phenotypes. Moreover, potential confounders such as dietary factors, inflammatory biomarkers, hormone levels, genetic predisposition, and history of joint injury were not included, which may bias the observed associations. From a clinical perspective, our findings suggest that METS-IR could serve as a convenient and low-cost tool to help identify individuals at higher risk of OA in both clinical and community settings, particularly where insulin assays are not routinely available. While METS-IR cannot replace diagnostic evaluation, it may complement existing clinical assessments by providing an additional layer of risk stratification. This may assist in guiding early preventive measures and in prioritizing individuals for further evaluation. Future studies with longitudinal follow-up and imaging-based confirmation are warranted to validate its predictive value and refine its role in clinical practice.

## 4. Conclusion

In this cross-sectional study of a US population, higher METS-IR levels were linearly and positively associated with OA prevalence. As a surrogate indicator of IR derived from routine metabolic parameters, METS-IR may serve as a valuable epidemiological tool for identifying individuals at increased risk of OA.

## Acknowledgments

Access to the NHANES data was provided by the National Center for Health Statistics (NCHS), Centers for Disease Control and Prevention (CDC).

## Author contributions

**Conceptualization:** Guibin Chen.

**Data curation:** Langming Li.

**Formal analysis:** Yanghua Weng, Hongdong Yang.

**Investigation:** Langming Li, Shuchi Lv.

**Resources:** Shuchi Lv.

**Supervision:** Guibin Chen.

**Validation:** Yanghua Weng, Hongdong Yang.

**Writing – original draft:** Yanghua Weng, Hongdong Yang.

**Writing – review & editing:** Yanghua Weng, Hongdong Yang, Langming Li, Shuchi Lv, Guibin Chen.

## Supplementary Material


