# Supplementary material for: Association between the metabolic score for insulin resistance and osteoarthritis prevalence: A cross-sectional population-based study
Source: Medicine (Baltimore). 2025 Oct 31;104(44):e44850. doi: 10.1097/MD.0000000000044850 (PMC12582681; doi:10.1097/MD.0000000000044850)
Supplement: Supplementary file 1 [file medi-104-e44850-s001.docx]

**Supplementary table 1 Sensitivity analysis of the association between METS-IR and osteoarthritis.**

|  |  | Model 1  OR (95%CI) P-value | Model 2  OR (95%CI) P-value | Model 3  OR (95%CI) P-value |
| --- | --- | --- | --- | --- |
| **Osteoarthritis** | METS-IR | 1.02 (1.01, 1.03) <0.001 | 1.02 (1.01, 1.03) <0.001 | 1.02 (1.01, 1.03) <0.001 |
|  | Q1 | [Reference] | [Reference] | [Reference] |
|  | Q2 | 1.27 (0.96, 1.69) 0.100 | 1.27 (0.94, 1.72) 0.110 | 1.32 (0.93, 1.88) 0.120 |
|  | Q3 | 1.22 (0.93, 1.58) 0.150 | 1.30 (0.96, 1.76) 0.086 | 1.28 (0.92, 1.77) 0.130 |
|  | Q4 | 1.73 (1.32, 2.28) <0.001 | 1.93 (1.44, 2.59) <0.001 | 1.71 (1.19, 2.44) 0.004 |
|  | P for trend | <0.001 | <0.001 | 0.003 |

CI: confidence interval; OR: odds ratio; Q: quartiles;

Model 1: no covariates adjusted; Model 2: adjusted for age, sex, and race; Model 3: adjusted for age, sex, race, educational level, PIR, smoke, drink, activity status, diabetes, CKD, CAD.
